# The c.863A>G (p.Glu288Gly) variant of the *CTSD* gene is not associated with CLN10 disease

**DOI:** 10.1002/mgg3.1777

**Published:** 2021-07-31

**Authors:** Juan Yang, Xiaoting Ding, Shasha Meng, Jinhua Cai, Weihui Zhou

**Affiliations:** ^1^ Chongqing Key Laboratory of Translational Medical Research in Cognitive Development and Learning and Memory Disorders National Clinical Research Center for Child Health and Disorders Ministry of Education Key Laboratory of Child Development and Disorders Children’s Hospital of Chongqing Medical University Chongqing China; ^2^ Department of Radiology of Children’s Hospital of Chongqing Medical University Chongqing China

**Keywords:** Cathepsin D, CLN10 disease, *CTSD*, neuronal ceroid lipofuscinoses, variation

## Abstract

**Background:**

Cathepsin D is a lysosomal aspartic protease encoded by the *CTSD* gene. It plays important roles in many biological processes. Biallelic loss‐of‐function mutation of *CTSD* is considered a cause of CLN10 disease. CLN10 is a rare autosomal recessive disorder that is one of 14 types of neuronal ceroid lipofuscinoses (NCLs). To date, only a few cases of CLN10 and 12 disease‐causing mutations have been reported worldwide.

**Methods:**

Exome sequencing was performed on a 15‐year‐old girl with pervasive brain developmental disorder. The effects of the identified variants were investigated through multiple functional experiments.

**Results:**

There were no differences in mRNA and protein expression, intracellular localization, maturation, and proteolytic activity between the cells with the mutant *CTSD* gene and those with the wild‐type *CTSD* gene.

**Conclusion:**

These results suggest that the c.863A>G (p.Glu288Gly) homozygous variant is not a pathogenic variation, but a benign variant.

## Graphical abstract

Here, we report the novel variant c.863 A>G (p.Glu288Gly) of the CTSD gene in homozygous form, identified in a patient with an NCL‐like disorder. The effects of the variant on CatD expression, maturation, cellular localization, and enzymatic activity were studied. These results suggest that the c.863A>G (p.Glu288Gly) homozygous variant is not a pathogenic variation, but a benign variant.

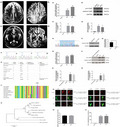

## 1 INTRODUCTION

Neuronal ceroid lipofuscinoses (NCLs), also known as Batten disease, are a group of severe and progressive neurodegenerative disorders that are inherited in a predominantly recessive manner and have mostly characterized by childhood onset (Williams & Mole, 2012). NCL disease is characterized by a gradual decline in vision and cognitive and motor function, together with seizures and dementia in the most common types of NCLs (Haltia & Goebel, 2013; Nita et al., 2016; Williams & Mole, 2012). There are 14 genetically distinct NCL disorders, denoted as CLN1 to CLN14. The NCL caused by defect of the *CTSD* gene is known as CLN10 disease (MIM # 610127). CLN10 disease is a rare type of NCL. To date, only nine reports worldwide have reported 12 rare mutations of the *CTSD* gene that caused NCL in 10 families.

The *CTSD* gene encodes protein Cathepsin D (CatD), which is an aspartyl proteinase in lysosome and is a member of the A1 family of peptidases. Human CatD is synthesized initially as pre‐pro‐cathepsin D and is then transported into the endoplasmic reticulum (ER) lumen (Nicotra et al., 2010; Zaidi et al., 2008). In the ER, the signal peptide is removed to generate inactive pro‐cathepsin D (pro‐CatD), which is then glycosylated and subsequently transported to the Golgi complex, where it is packaged into budding transport vesicles and delivered to late endosomes. In the endosomes, the pro‐peptide (44 aa) is removed from pro‐CatD (53 kDa) to generate an active intermediate single‐chain molecule (48 kDa). The latter eventually undergoes another proteolytic process in the lysosomal compartment to be converted to a mature form of two chains, namely the N‐terminal 14‐kDa light chain and the C‐terminal 34‐kDa heavy chain (Benes et al., 2008; Minarowska et al., 2008; Zaidi et al., 2008).

CatD is ubiquitously expressed in all cells in human tissues and is highly expressed in the brain (Vidoni et al., 2016). Through its protease activity, the main functions of CatD include degradation of unfolded or abnormal intracellular proteins, activation of hormones and growth factors, and participation in many biological processes such as cellular protein renewal and tissue homeostasis (Benes et al., 2008; Vidoni et al., 2016). In addition to its proteolytic function in lysosomes, CatD has been shown to promote the invasion and proliferation of cancer cells (Nomura & Katunuma, 2005), participate in the apoptotic pathway that is not related to caspase (Bröker et al., 2005; Liaudet‐Coopman et al., 2006), and play a unique role in autophagy (Marques et al., 2020).

Here, we report the novel variant c.863 A>G (p.Glu288Gly) of the *CTSD* gene in homozygous form, identified in a patient with an NCL‐like disorder. The effects of the variant on CatD expression, maturation, cellular localization, and enzymatic activity were studied.

## 2 METHODS

### 2.1 Subject

A 15‐year‐old girl with pervasive brain developmental disorder and her unaffected parents were recruited into this study. Blood samples were collected from the patient and her parents.

### 2.2 Variant detection

Targeted clinical exome sequencing was performed with genomic DNA extracted from the subjects’ peripheral blood; the sequencing was performed by MyGenostics, Beijing, China. Sanger sequencing was performed with PCR products to amplify the *CTSD* gene from genomic DNA with the primers CTSD‐1F (5′‐TCCATCCCCACATCCCTCTG) and CTSD‐1R (5′‐GCTTGTAGCCTTTGCCTCCC). Localization of variant is based on the reference sequence NM_001909.5. Sequence conversation analysis was performed with the ClustalW program to align the amino acid sequences downloaded from different species in the NCBI database. The evolutionary history was inferred using the neighbor‐joining method with MEGA7.0.

### 2.3 Plasmid construction

The expression plasmid pcDNA3.1+/C‐(K)‐DYK (Genscript) was linearized by cleavage using the restriction enzymes *Kpn*I and *Apa*I. The cDNA sequences of wild‐type and mutant c.863 A>G in the human *CTSD* gene were amplified by PCR with the cDNA library of white blood cells as the templates from normal control and the patient, respectively. The primer sequences used for PCR were pCTSD‐F (5′‐TTTAAACTTAAGCTTGGTACGCCACCatgcagccctccagccttctgcc) and pCTSD‐R (5′‐TCGTCGTCATCCTTGTAATCgaggcgggcagcctcggcgaag). The PCR products were ligated with the linearized plasmid pcDNA3.1+/C‐(K)‐DYK by homologous recombination according to the ClonExpress MultiS One Step Cloning Kit manual (Vazyme Biotech) and were named pcDNA3.1‐CTSDWT‐DYK(CD‐WT+flag) for the wild‐type and pcDNA3.1‐CTSD863M‐DYK(CD‐863M+flag) for the mutant. The inserted cDNA of the human *CTSD* gene in both plasmids was tagged with a flag sequence at the 3‐prime end of the cDNA. To construct the plasmids without a flag tag, DNA fragments were amplified with primer pairs (pCTSDRT‐2F: 5′‐TGATAAACCCGCTGATCAGCCTCGACTGTGC‐3’ and pCTSDRT‐2R: 5′‐gaggcgggcagcctcggcgaag‐3′) and the plasmids pcDNA3.1‐CTSDWT‐DYK or PCDNA3.1‐CTSD863M‐DYK as templates. The resulting PCR products were blunted and self‐ligated to generate plasmids pcDNA3.1‐CTSDWT(CD‐WT) and pcDNA3.1‐CTSD863M(CD‐863M). The cDNA sequences of the *CTSD* gene in the four plasmids were compared with the reference sequence of NM_001909.5 and confirmed by DNA sequencing.

### 2.4 Cell culture and transient transfection

Human embryonic kidney 293T (HEK293T) cells from ATCC were cultured in Dulbecco's modified eagle's medium (Gibco) supplemented with 10% fetal bovine serum (Gibco) and 1% penicillin/streptomycin at 37℃ in an incubator containing 5% CO₂. The cells were grown to approximately 70%–90% confluence before transfection and were continually cultured for 12–24 h after transient transfection. Transfection of different plasmids was carried out using Lipofectamine 2000 (Invitrogen) according to the supplier's instructions.

### 2.5 Extraction of total RNA and reverse transcription

Cells in 6‐well plates were transfected with 3 μg of plasmid DNA per well and cultured for 24 h. The total RNA was isolated using the improved one‐step method of guanidinium isothiocyanate and phenol (Bioteke), according to the manufacturer's protocol. DNase I (TaKaRa) was used to treat 1 μg of total RNA in order to completely remove residual DNA in the RNA samples. The first‐strand cDNAs were synthesized using a PrimeScript RT Reagent Kit with gDNA Eraser (TaKaRa).

### 2.6 Quantitative real‐time PCR (qPCR)

qPCR was performed with the following reagents: 2×QuantiNova SYBR Green PCR Master Mix (QIAGEN), CTSD primers CTSDRT‐1F (5′‐GCCCCGTCTCAAAGTACTCC‐3′) and CTSDRT‐1R (5′‐TGGATCCAGCAAGCGATGTC‐3′), and GAPDH primers 5′‐CTCCTCCACCTTTGACGC‐3′ and 5′‐CCACCACCCTGTTGCTGT‐3′. For each sample, triplicates of each primer set were analyzed, and the relative mRNA expressions of the *CTSD* gene were calculated using the comparative CT method normalized by GAPDH.

### 2.7 Western blotting analysis

Cells in six‐well plates were transfected with 3 μg of plasmid DNA per well. 24 h after transfection, the cells were harvested and lysed in 100 µl of cell lysis buffer (Beyotime) supplemented with protease inhibitors (Roche) for 30 min on ice. Cell lysates were obtained by centrifugation at 14,000× *g* for 15 min at 4℃. A BCA protein assay (Thermo Fisher Scientific) was used to measure the concentration of the protein in the cell lysates. The cell lysates were separated on 4%–12% SDSTris‐glycine gel, transferred onto PVDF membranes, and blotted with primary and secondary antibodies. The expressed proteins were visualized by the Odyssey system (LI‐COR Biosciences) and quantified using Quantity One. The relative amounts of the proteins were normalized with control proteins. The antibodies against human Cathepsin D (Abcam, ab6313), β‐actin (Sigma, A5441), and GAPDH (Affinity Biosciences, AF7021) were used as the primary antibodies, IRDye‐labeled anti‐mouse (Odyssey IRDye 880CW, 926‐32220) and anti‐rabbit IgG antibodies (Odyssey IRDye 680, 926‐32221) were used as the secondary antibodies.

### 2.8 Construction of *CTSD* knockout cell strain

The oligonucleotides CTSD‐TOP‐100 (5′‐ACCGACAAGTCACGTCATCCATCCGC‐3′) and CTSD‐BOTTOM‐100 (5′‐AAACGCGGATGACGACGACGACGATGTCGTC‐3′) were annealed and then ligated with linearized plasmid pSpCas9(BB)‐2A‐GFP(PX458), which was digested by restriction endonuclease *Bpi*I, to generate plasmid CTSD‐PX458‐Sg100. To knockout the *CTSD* gene, the plasmid CTSD‐PX458‐Sg100 was transiently transfected into HEK293T cells. 24 h after transfection, the cells were harvested, GFP‐positive cells were sorted into 96‐well plates with flow cytometry, with a single cell per well to form the single‐cell strain. Once the cells in each well had grown in sufficient numbers, PCR was performed using the primer pairs CTSD‐Sg100‐F (5′‐ttcactgacttgggggagact‐3′)/CTSD‐Sg100‐R (5′‐agaaaggagtgtggctgagc‐3′) and genomic DNA extracted from the single‐cell strain as the template. The resulting PCR product was sequenced to screen *CTSD* gene knockout cell strains. Western blotting was used to verify that the cells no longer expressed the CatD protein.

### 2.9 Immunofluorescence assay

The *CTSD* gene knockout cells were grown on coverslips placed in the wells of a 12‐well plate and were transfected with 1.5 μg plasmid DNA per well. Twenty‐four hours after transfection, the cells were washed with PBS three times and then incubated in 4% paraformaldehyde in PBS for 30 min to fix the cells. The cells were then washed again three times with PBS, then incubated with PBS containing 0.1% Triton X‐100 for 30 min to permeabilize the cells. After permeabilization, the cells were washed three times with PBS and blocked with 5% BSA for 1 h. Subsequently, the permeabilized cells were incubated with the corresponding primary antibody overnight at 4°C in a shaker. On the second day, the cells were washed with PBS three times, and the corresponding secondary antibody conjugated with Alexa Fluor 488 or 546 was added; they were then incubated at room temperature for 1 h in the dark and then washed three times again with PBS. Finally, the coverslips were mounted on a slide loaded with fluorescent quencher and observed with a Nikon C2+ laser confocal microscopy imaging system. NIS‐Elements software was used to analyze the fluorescence pictures. Anti‐Cathepsin D antibody (Abcam, ab75852), LAMP1 (D4O1S) mouse mAb (Cell Signaling Technology, #15665), and PDI monoclonal antibody (RL90) (Invitrogen, MA3‐019) were used as the primary antibodies. Donkey anti‐rabbit IgG (H+L) highly cross‐adsorbed secondary antibody, Alexa Fluor Plus 488 (Invitrogen, A32790), and goat anti‐mouse IgG (H+L) cross‐adsorbed secondary antibody, Alexa Fluor 568 (Invitrogen, A‐11004) were used as the secondary antibodies.

### 2.10 CTSD enzymatic activity assay

Cells were seeded in six‐well plates and transfected with 3 μg of the plasmids. Twenty‐four hours after transfection, 1×PBS was used to wash the cells once and the cells were resuspended. After centrifuging the samples for 5 min at 4°C, the cell pellet was treated with Cell Lysis Buffer by incubating the cell lysates on ice for 10 min. The clear cell lysates were harvested by centrifuging at 4°C. A CTSD activity assay (Abcam, ab65302) was performed according to the supplier's protocol.

## 3 RESULTS

### 3.1 Clinical profile

**FIGURE 1:**
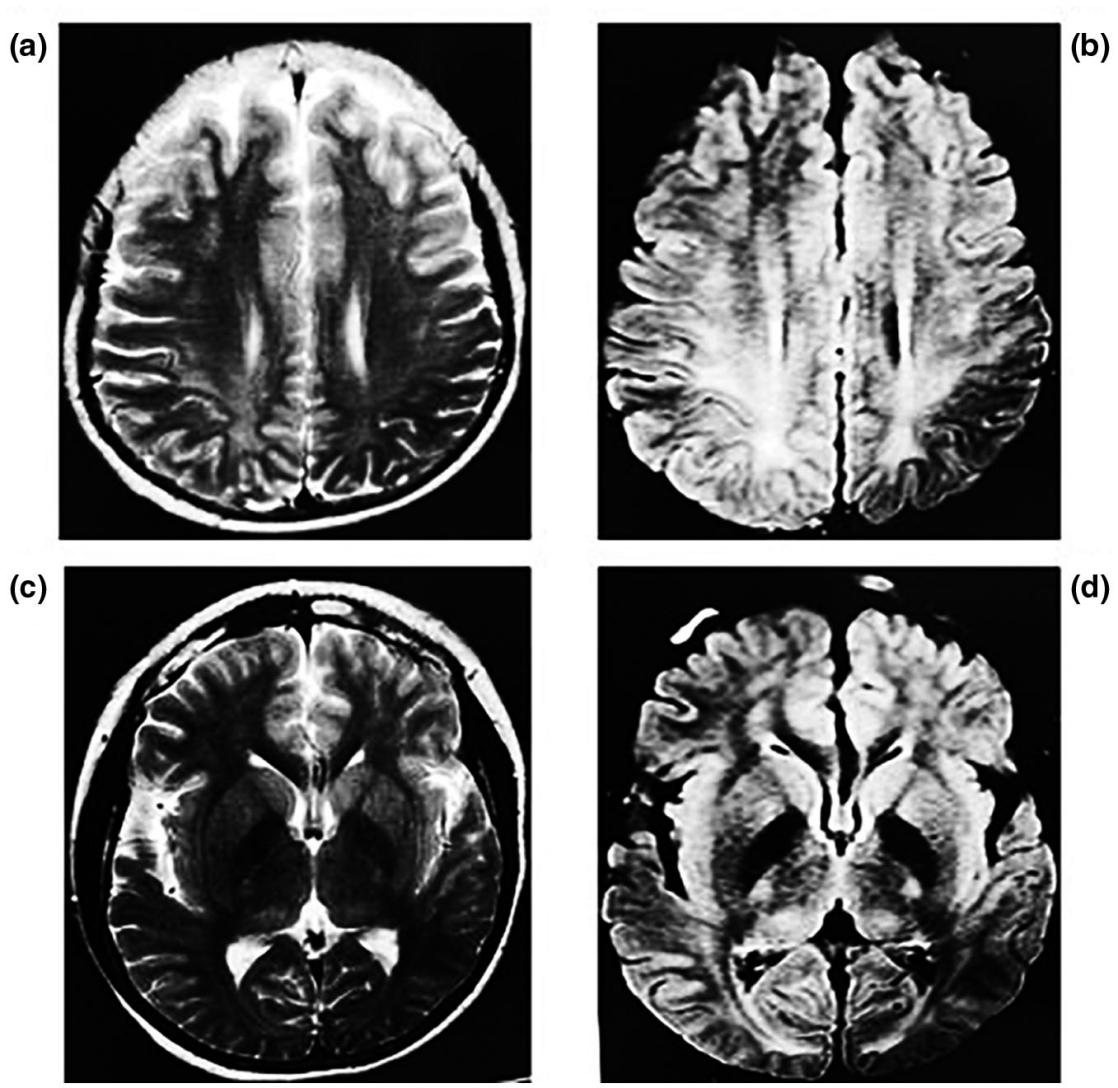
Magnetic resonance imaging (MRI) of the brain. (a and b) T2‐weighted and T2 FLAIR images present bilateral symmetrical patchy hyperintensity in the white matter of the parietal lobe. (c and d) T2‐weighted and T2 FLAIR images show symmetrical flaky hyperintensity in the bilateral thalamus

The patient was a 15‐year‐old girl who was referred to the clinic due to developmental delay and mental retardation. She presented with signs of dementia, absent speech, slow walking, poor reactions, and severely reduced IQ (IQ 40); she had no special facial features. Magnetic resonance imaging (MRI) revealed a wide range of abnormal signals in the cerebrum and thalamus suggestive of demyelination of cerebral white matter; however, the shape, size, and location of the ventricles were within the normal range (Figure 1). At the age of three, she was noted as speechless. After that, she exhibited progressive retardation of development and intellect. Before the age of seven, she suffered from frequent generalized convulsions. In consideration of the clinical features, course of illness, and MRI findings, the diagnosis of pervasive brain developmental disorder of uncertain cause was made.

### 3.2 Identification of a rare homozygous missense variant of the *CTSD* gene

**FIGURE 2:**
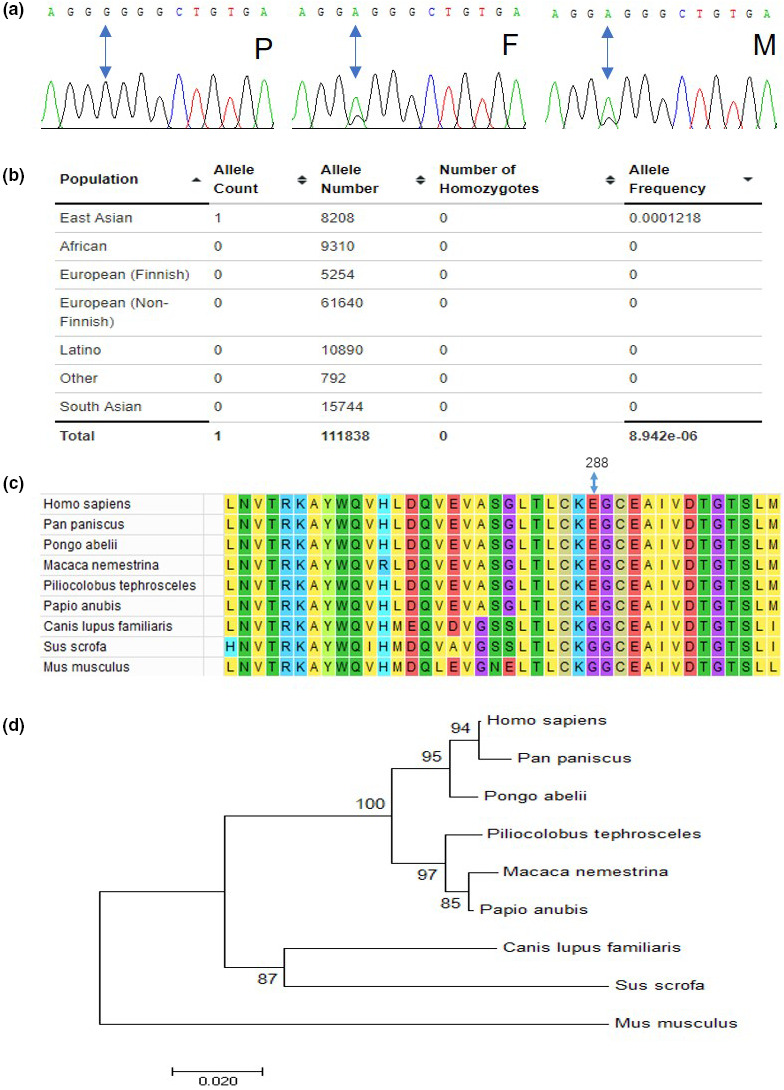
Identification of a rare homozygous mutation. (a) DNA sequencing identified a homozygous variant in the patient (P) and heterozygous variants in the patient's father (F) and mother (M), respectively. (b) The allele frequency of the variant is less than 0.0001 in the East Asian population. (c) Alignment of polypeptide sequences (including residue p.E288) in different species. Localization of variant is based on the reference sequence NM_001909.5. (d) Evolutionary relationship in these species

Since the clinical manifestations and MRI workup failed to suggest any underlying cause, a clinical exome sequencing was performed. The sequencing results revealed six suspicious variants in six genes, including *TUBGCP5*, *GATAD2B*, *KMT2D*, *HDAC4*, *DOCK8*, and *CTSD*. The variant c.863A>G in the *CTSD* gene is the only homozygote and the other five variants are all heterozygotes. In combination with the patient's clinical presentation, which is similar to that of NCL disease, and given the causal role of *CTSD* gene defect in the pathogenesis of NCL disease, the variant c.863A>G of the *CTSD* gene was preferentially studied for its possible effects. First, the patient's homozygosity for the variant was confirmed by Sanger sequencing and subsequent Sanger sequencing of the parents’ genomic DNA from white blood cells revealed that both were heterozygote carriers of the same variant (Figure 2a). The variant c.863A>G is located in chr11‐1775333 (GRCh37) and the SNP rs number is 773273362. The variant c.863A>G of the *CTSD* gene was predicted to lead to the substitution of glycine for glutamic acid at amino acid position 288 (p.Glu288Gly or p.E288G) of the CatD protein. To explore the frequency of the variant in the population, a search of the Exome Aggregation Consortium (ExAC), an exome variant database, was performed. Among the total of 111,838 alleles, only one in heterozygous form was found in an East Asian population of 4104 people (Figure 2b). Thus, c.863A>G in the *CTSD* gene is a rare and missense variant. Sequence conservation analysis revealed that the amino acids around residue E288 are highly conserved among closely related species, but this is not the case for distant species on the evolutionary tree, such as mice and dogs (Figure 2c,d).

### 3.3 The variant does not decrease CatD expression in cells

**FIGURE 3:**
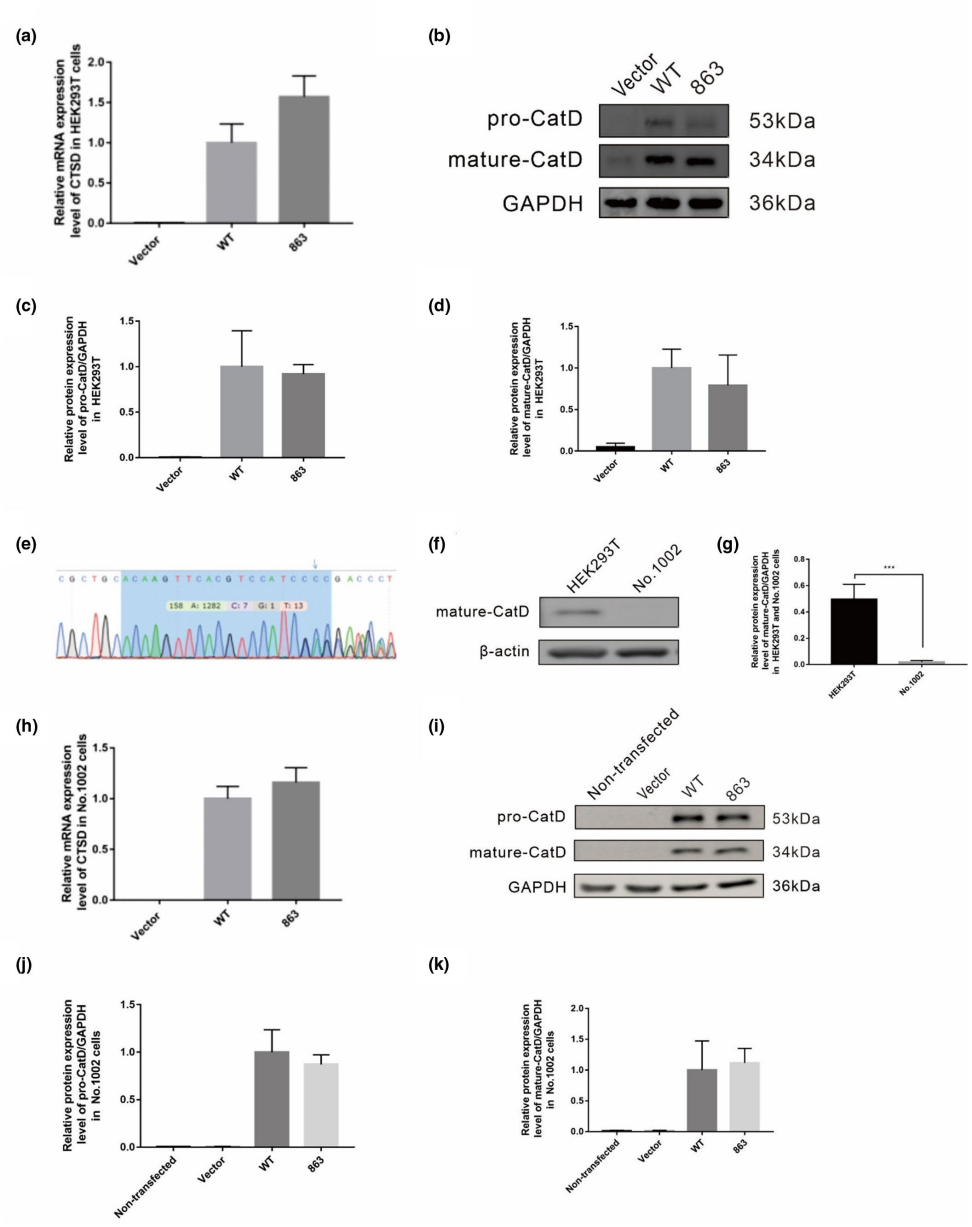
The variant does not decrease CatD expression in cells. HEK293T cells (a‐d) and No. 1002 cells (h‐k) were transfected with plasmids of blank vector, CD‐WT, or CD‐863M. (a) The mRNA level of the *CTSD* gene was detected by qPCR in HEK293T cells. (b) The protein level of the *CTSD* gene was detected by Western blot in HEK293T cells. (c and d) There were no differences in pro‐CatD (c) and CatD (d) between the wild‐type and 863 mutation in HEK293T cells. All values above are expressed as means ± SD, N=3. One‐way ANOVA was used in this experiment. (e) Sequencing peak diagrams of No. 1002 cells. Blue regions represent DNA sequences targeted by SgRNA sequences. The arrow indicates the first base changes due to non‐homologous repair. (f) Verification of the endogenous CatD protein expression of No. 1002 cells and HEK293T cells at the protein level. HEK293T cells were used as control cells. (g) CatD protein is almost non‐existent in No. 1002 cells. All values above are expressed as means ± SD, *N* = 3. ***p < 0.001 by t‐test. (h) The mRNA level of the *CTSD* gene was detected by qPCR in No. 1002 cells. (i) The protein level of the *CTSD* gene was detected by Western blot in No. 1002 cells. (j and k) No differences in pro‐CatD (j) and CatD (k) between the wild‐type and 863 mutation in No. 1002 cells. All values above are expressed as means ± SD, *N* = 3. One‐way ANOVA was used in this experiment

Pathogenic nucleotide variations often affect the expression of the mRNA or protein that the gene encodes. To evaluate the effect of the variant c.863A>G on the expression of the *CTSD* gene, eukaryotic expression plasmids containing the coding sequences of wild‐type or mutant human *CTSD* genes were constructed and transiently transfected into HEK293T cells. The mRNA and protein levels of the *CTSD* gene in cells transfected with the plasmids were detected by qPCR and Western blotting, respectively. The results showed that the variant did not cause a decrease in mRNA levels of the *CTSD* gene (Figure 3a). The protein levels of the *CTSD* gene, both in the immature form of pro‐CatD and the mature heavy chain, did not differ between cells transfected with wild‐type expression plasmids and those transfected with mutant expression plasmids (Figure 3b–d).

Since the *CTSD* gene is extensively expressed in various tissues and cells in the human body, in order to avoid interference by endogenous CatD proteins in the experimental cells, CRISPR Cas9 technology was applied to knock out the *CTSD* gene of HEK293T cells. Sanger sequencing showed that base substitution occurred in the coding sequence of the *CTSD* gene targeted by the specific small guide RNA (SgRNA) sequence and caused subsequent base‐pairing errors, which caused frameshift mutations (Figure 3e). Western blotting was applied to verify the protein expression of the *CTSD* gene in the knockout cell strain. The results showed that the *CTSD* gene knockout cells had strong expression of endogenous mature CatD proteins while the knockout cell strain No. 1002 had almost no expression of mature proteins (Figure 3f,g).

The plasmids CD‐WT or CD‐863M were transfected into the *CTSD* gene knockout cell strain No. 1002. The mRNA expression of the *CTSD* gene was measured by qPCR and the results showed that there was no difference in the mRNA levels of the mutant and wild‐type (Figure 3h). Next, the level of CatD protein that exogenously expresses wild‐type or mutant *CTSD* gene in No. 1002 cells were examined. The results showed that there was almost no endogenous protein expression in the cells transfected with the blank vector (Figure 3i–k). Cells transfected with the plasmids containing the wild‐type *CTSD* gene showed strong expression, both in the immature form of pro‐CatD and the mature‐CatD protein, while cells transfected with the plasmids containing mutant *CTSD* showed similar protein levels (Figure 3i–k). This result is consistent with that found in HEK293T cells.

### 3.4 The variant does not alter the normal intracellular transport of the CatD protein

**FIGURE 4:**
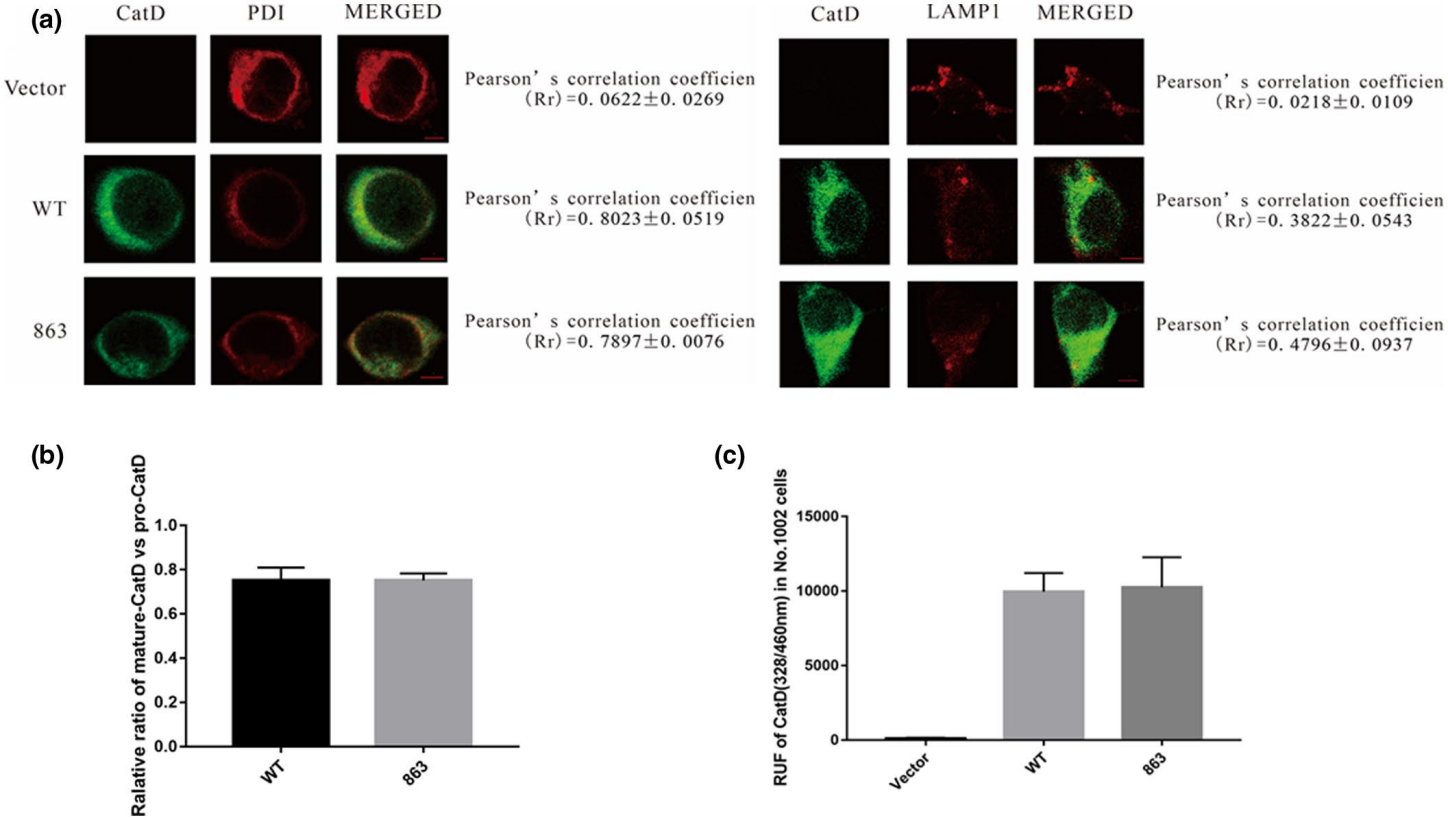
The intracellular localization of CatD and enzyme activity in No. 1002 cells. (a) The immunofluorescence cell localization of wild‐type and mutant CatD expressed in No. 1002 cells. After transfecting vector, wild‐type, and mutant plasmids into No. 1002 cells, the co‐localization of the CatD protein (green) and lysosome (red), as well as the co‐localization of the CatD protein (green) and endoplasmic reticulum (red) were observed. (b) The ratio of mature‐CatD and pro‐CatD in No. 1002 cells transfected with the wild‐type and mutant of the *CTSD* gene, respectively. (c) The CatD activity of wild‐type and mutant No. 1002 cells. CatD activity expressed as relative fluorescence units (RFU) per million cells

As mentioned in the introduction, the maturation of the CatD protein depends on its normal transport among organelles within the cell. Since there was no difference in total protein level between the mutant and the wild‐type of the *CTSD* gene, immunofluorescence was applied to investigate whether the mutant could cause differences in the expression and localization of the CatD protein in the ER and lysosome of cells. In order to accurately track the transportation of mutated CatD protein within cells and to avoid interference by endogenous CatD proteins within cells, the plasmids CD‐WT or CD‐863M were transiently transfected into knockout strain No. 1002 cells. The cells were co‐immunolabeled with anti‐CatD antibody and either anti‐LAMP1 or anti‐PDI antibody and were double‐stained with different fluorescent secondary antibodies. The results showed that the cells transfected with the CD‐WT or CD‐863 M plasmid had similar staining patterns (Figure 4a). In both cells, the CatD protein was co‐localized with the ER protein marker of protein disulfide isomerase (PDI) and the lysosome protein marker of lysosomal‐associated membrane protein 1(LAMP1), respectively, suggesting that the wild‐type and mutant proteins had the same intracellular trafficking. In addition, the relative ratios of the heavy chain to the immature form of pro‐CatD in both cells transfected with the CD‐WT or CD‐863M plasmid were calculated based on the optical densities of the bands in the Western blot analysis in Figure 3f. This ratio also partially reflects the status of intracellular trafficking of the CatD protein. As shown in Figure 4b, the ratios were the same for the two cells transfected with either the wild‐type or mutant *CTSD* gene. Taken together, these results indicate that the variant c.863A>G does not affect the normal transport of the CatD protein in cells.

### 3.5 The variant does not change the enzyme activity of the CatD protein

Since the main function of the CatD protein is to act as a lysosome protease that cleaves and degrades unfolded or abnormal intracellular proteins to maintain cellular homeostasis, we investigated whether the variant c.863A>G in the *CTSD* gene had any effects on the enzymatic activity of the CatD protein. The enzymatic activity of cell lysates from the *CTSD* gene knockout cell strain No. 1002 transfected with the CD‐WT or CD‐863M plasmid was measured. The results showed that the cells transfected with blank vector had extremely low values, suggesting that there was almost no endogenous enzyme activity in the knockout cell strain, which is consistent with the Western blotting results. The cells exogenously expressing the wild‐type and mutant *CTSD* gene showed significantly higher enzymatic activity than that of the blank vector, but there was no significant difference between the mutant and wild‐type (Figure 4c). Therefore, the variant c.863A>G does not alter the enzyme activity of the CatD protein.

## 4 DISCUSSION

Targeted exome sequencing of a patient with pervasive brain developmental disorder was performed in the present study and a homozygous variant c.863A>G in the *CTSD* gene was identified. The variant c.863A>G is located in exon 7 of the *CTSD* gene and is a missense variation leading to the substitution of glycine for glutamic acid at amino acid position 288 (p.E288G) of the CatD protein. The CatD protein has two key catalytic sites of aspartic acid at positions 97 and 295 (Baldwin et al., 1993). The residue 288 is very close to one of them at position 295, suggesting it has a potential effect on function (Baldwin et al., 1993). This variant is novel and rare since it has not been reported previously in the literature and only one allele was found in the ExAC database. Analysis of sequence conservation indicated that the amino acids surrounding residue E288 are highly conserved among closely related species. At first, it was suspected that this might be a pathogenic mutation. To verify this hypothesis, a series of functional tests were performed. However, no differences in enzyme activity, transcription level, protein level, and intracellular localization between the mutant and wild‐type were observed. All of the results indicated that the variant did not cause damage to its function.

Biallelic mutations of the *CTSD* gene leading to NCL or NCL‐like disease have been reported in nine studies (Doccini et al., 2016; Fritchie et al., 2009; Hersheson et al., 2014; Meyer et al., 2015; Regensburger et al., 2020; Siintola et al., 2006; Steinfeld et al., 2006; Thottath et al., 2019; Varvagiannis et al., 2018), in which, 12 different mutations were identified from 10 families in total. Among those mutations, 4 of them were insertion or deletion mutations causing amino acid frameshift, and the other 8 were missense mutations. In the original literature, all pathogenic mutations tested for their effect on enzyme activity resulted in a decrease in enzyme activity, ranging from 0% to 26%. Moreover, the overall severity of the disease appeared to be positively correlated with the degree of enzyme activity loss. Bunk et al. tested the effects of six of these pathogenic variants causing NCL disease on the expression, intracellular localization, maturation, and enzyme activity of the CatD protein using consistent experimental methods and conditions. The authors reported that all six variants resulted in a loss or significant reduction of enzyme activity (Bunk et al., 2021). In addition to these pathogenic mutations that cause NCL disease, there are several other variants of the *CTSD* gene thought to be associated with other diseases, such as Parkinson's disease and Alzheimer's disease (Bunk et al., 2021). Excessive burden of lysosomal storage disorder gene variants was observed in Parkinson's disease while genetic polymorphism of CatD was strongly associated with the risk for developing sporadic Alzheimer's disease. Moreover, a positive association between the CatD Ala224Val gene polymorphism and the risk of Alzheimer's disease has been reported, but functional experiments have shown that these variants do not affect intracellular localization, maturation, or enzymatic activity of the CatD protein (Bunk et al., 2021). It has also been reported that the potential subtle effects of the c.C224T polymorphism (p.Ala58Val) of the *CTSD* gene on lysosomal function may not be associated with childhood‐onset neurodegenerative diseases (Kettwig et al., 2018). The variant found in the current study does not cause a decrease in enzyme activity. Moreover, the MRI examination of our case differs from previous reports. In our case, there was no obvious evidence of brain atrophy, but there were extensive abnormal signals in the brain and thalamus, suggesting demyelination of the central nervous system. Central nerve demyelination can cause dementia and movement disorders in humans. Based on the above results, it is concluded that the novel variant c.863A>G in the *CTSD* gene might not be a pathogenic mutation implicated in CLN10 disease, but a benign variant. It may be that other undiscovered mutations of other genes caused the patient to develop NCL‐like symptoms.

The diagnosis of NCL is usually based on age of onset, initial clinical symptoms, course of clinical progression, neuroimaging, and pathological findings. The common clinical features of NCLs are progressive cognitive and motor decline, visual impairment, epileptic seizures, and neuroimaging abnormalities such as diffuse brain atrophy, especially cerebellar atrophy, and thalamic hypointensity and white matter hyperintensity on brain MRI (Kamate et al., 2021; Nita et al., 2016). However, these clinical manifestations of NCL are not specific, as other diseases such as leukodystrophy, peroxisomal disorders, lysosomal storage disorders, and mitochondrial diseases may also exhibit overlapping manifestations of NCLs (Nita et al., 2016). In addition, fourteen different NCLs are clinically and genetically heterogeneous (Schulz & Kohlschutter, 2013). All this makes the diagnosis complicated and difficult (Setty et al., 2013; Wiśniewski et al., 1997). Precise diagnosis requires electron microscopy of skin biopsy, enzyme measurement for some types of NCLs, and/or the use of next‐generation sequencing technology (Hersheson et al., 2014; Kamate et al., 2021). Our case lacked obvious visual impairment but had a unique inability to speak. Although CLN12 disease also exhibited dysarthric speech (Nita et al., 2016), exome sequencing did not reveal rare pathogenic variants in the *ATP13A2* gene and twelve other NCL genes. Therefore, the accurate diagnosis and etiology of our case require further investigations.

In conclusion, a benign missense variant in the *CTSD* gene was identified in a patient with generalized brain development disorder. The confirmation of this site has significance for the expansion of the NCL mutation database.

## CONFLICT OF INTEREST

The authors declare that they have no competing interests.

## AUTHORS’ CONTRIBUTIONS

W.Z. conceived and designed the research, wrote the manuscript; Y.J., X.D., S.M. performed experiments and wrote the manuscript; J.C. helped in analyzing the result of MRI.

## CONSENT FOR PUBLICATION

All authors approve of this publication.

## 5 Ethical compliance

This study was approved by the ethics committee of the Children's Hospital of Chongqing Medical University. All experimental procedures were carried out in accordance with the approved guidelines and relevant regulations. Written informed consent was obtained from the patient's parents.

## Data Availability

The datasets used and/or analyzed during this study are available from the corresponding author on reasonable request.
